# Evaluation of outcomes in 201 child and youth survivors of Child Sexual Abuse (CSA) who participated in a one-year multimodal treatment program

**DOI:** 10.1371/journal.pone.0354692

**Published:** 2026-07-23

**Authors:** Matthew Reeson, Hannah Pazderka, Wanda Polzin-Homan, Yifeng Wei, Andrew Greenshaw, Peter H. Silverstone

**Affiliations:** 1 Department of Psychiatry, University of Alberta, 1E1 Walter Mackenzie Health Sciences Center (WMC), Edmonton, Canada; 2 Little Warriors Be Brave Ranch, Sherwood Park, Alberta, Canada; University of Toronto, CANADA

## Abstract

**Background and rationale:**

Child sexual abuse (CSA) is a pervasive and prevalent form of early-life trauma that can increase the risk for PTSD, mood disorders, substance abuse, harmful behaviour, non-suicidal self-harm, suicidal ideation, and death by suicide. Preliminary research has shown positive impacts of multimodal treatment for youth survivors of CSA, but no ‘gold-standard’ currently exists for CSA treatment. The aim of this study was to provide a secondary analysis of data from a longitudinal, episodic, and multimodal treatment program specifically designed to treat child and adolescent survivors of CSA.

**Methods:**

Child and youth survivors of CSA aged 8–17 completed a one-year multimodal treatment program at a dedicated facility known as the Little Warriors Be Brave Ranch. Participants completed self-report outcome measures for PTSD, depression, anxiety, self-esteem, quality of life, family functioning, and substance use at intake and graduation from the program. Changes in reported outcomes for child and youth participants were analyzed over the course of treatment.

**Results:**

A total of 201 sequential individuals who completed the one-year treatment program between January 2022-January 2026, from an initial group of 253 participants, were included in this analysis. Most of the completers were biologically female (n = 166, 82.6%) and were adolescents aged 13–17 (n = 116), while the remainder were children aged 8–12 (n = 85). Overall, positive changes in all outcomes were reported in participants from both groups. The largest effect was reported in adolescent PTSD symptoms (d = 0.94, p < 0.001), depression (d = 0.88, p < 0.001), and anxiety (d = 0.86, p < 0.001). Large effect sizes were also reported in levels of PTSD (d = 0.72, p < 0.001), depression (d = 0.82, p < 0.001), and anxiety (d = 0.64, p < 0.001) in child participants. Participants whose PTSD scores remained above the clinical threshold at graduation had higher levels of family dysfunction, more complex histories of trauma, and lower resilience scores.

**Conclusion:**

The findings of this study support the use of a multimodal treatment program for youth CSA survivors. The results suggest that this program design has the capacity to benefit CSA survivors across several domains of mental health and well-being. However, the findings of this study are limited by the lack of a control group. Future research should investigate the long-term effects of treatment, and include comparisons to a control sample, to further elucidate the sustainability of these positive therapeutic outcomes.

## Introduction

Traumatic experiences are stressful events that overwhelm an individual’s coping capacity, triggering physiological and psychological responses. These experiences can lead to long-term effects such as emotional dysregulation, cognitive impairments, interpersonal difficulties, harmful behaviors (e.g., substance abuse), sleep disturbances, and post-traumatic stress disorder (PTSD) [1,2]. Early-life trauma is particularly impactful because the developing brain is highly plastic, making overwhelming stressors capable of inducing lasting changes [3]. Studies on adverse childhood experiences (ACEs) reveal associations with chronic disease, psychological distress, PTSD, depression, anxiety, reduced self-esteem, substance use disorder, self-harm, and suicidal ideation [4,5].

Child sexual abuse (CSA) is among the most severe early-life traumas and remains globally pervasive [6]. CSA generally refers to non-consensual sexual activity involving individuals under 18,including exposure to pornography, genitalia, sexual acts, oral-genital contact, attempted intercourse, or penetration [7,8]. To complicate matters, technology has expanded CSA risks, as perpetrators exploit social media (e.g., Snapchat, Instagram) and gaming platforms (e.g., Roblox, Minecraft) to groom youth [9]. Artificial Intelligence technology now enables the creation of synthetic sexual content featuring minors, prompting policy makers to replace “child pornography” with “Child Sexual Abuse Material (CSAM)” [10]. A number of studies have attempted to determine the worldwide prevalence of CSA, but inconsistent operational definitions and differences in methodologies has resulted in varied results. Nonetheless, global estimates approximate that roughly 1 in 5 girls and 1 in 12 boys will experience some form of sexual abuse prior to adulthood [6,11]. It has been suggested that, given reporting issues and varied rates of disclosure, these estimates may significantly underestimate the prevalence of CSA [12,13]. Research in CSA prevalence has also found a disproportionate impact on individuals from minority populations, such as the LGBTQ2 S+ community and Indigenous Canadians [14,15]. The advancement of online technologies has only increased the risk and likelihood for children to be abused sexually [16]. As such, prevention strategies need to be adopted and best-practice treatment options for survivors need to be developed, implemented, and evaluated.

At present, several different psychological approaches have been proposed for CSA treatment. Metanalyses on best-practice treatment options consider Trauma-Focused Cognitive Behavioural Therapy (TF-CBT) as the most effective current treatment modality for CSA survivors [17]. TF-CBT uses a trauma-informed approach to cognitive behavioural modalities and integrates specific considerations for the complex effects of early-life trauma [18]. Several other approaches, including present-focused and trauma-focused group interventions may also have a positive effect on psychological outcomes [19]. Other modailies have shown evidence for improved treatment outcomes, including eye movement desensitization and reprocessing (EMDR) [20], art and expressive therapies [21], animal-assisted therapy [22], recreational and play therapy [23], psychosocial interventions [24], and structure therapeutic milieu [25]. Given the diversity of genetic predispositions, environmental influences, and nature of the traumatic experience(s), the effects of CSA can manifest heterogeneously, making it difficult to develop an approach that can address this diversity. Research suggests that combination or multimodal therapy may be the best approach and provide the optimal outcomes for this population [26]. Further investigation into the effect of multimodal treatment approaches on youth survivors of CSA has shown preliminary positive impacts on symptoms of PTSD, depression, and anxiety, as well as improvements in self-esteem and quality of life [27,28].

One major factor that requires consideration in the treatment of youth survivors of trauma is the integration of the child’s caregiver into the treatment process [29]. In trauma work, trauma histories of the child or adolescent’s caregiver can dramatically impact the treatment process. Many non-offending caregivers may react to their child’s abuse through blame or shame, which can become a barrier for the child’s access to treatment [30]. Research has shown that a caregiver’s personal experience of trauma can dramatically impact their emotions and their beliefs towards their child’s trauma and its impacts [31]. A child may be able to learn coping strategies or cognitive tools in treatment, but if a caregiver fails to facilitate the maintenance of these skills, the effects of treatment may be mitigated.

To address this issue we developed the Four Stories approach to trauma treatment, as outlined by Polzin et al. [32]. This approach suggests four primary aspects that need to be considered when moving families past the effects of trauma: (1) The child’s past narrative; (2) the child’s present narrative; (3) the caregiver’s past narrative; (4) the caregiver’s present narrative. Both the caregiver and child may influence treatment outcomes by remaining in a past-orientation focused on the trauma. The goal is to move both child and caregiver into a present-orientation that focuses on posttraumatic growth and healing. This can be done by providing caregivers with self-directed psychoeducation on the impacts of trauma and parenting strategies, remote or in-person parent coaching sessions with dedicated family therapists, or intensive family therapy treatment aimed at integrating dyadic treatment into the child’s therapeutic program.

The treatment program under investigation in this study integrated the Four Stories approach into a complex 1-year multimodal program to treat survivors of CSA. This program combines multiple evidence-based modalities, in addition to the Four Stories framework, to treat youth CSA survivors. By analyzing self-report outcome data on domains of mental health, well-being, and family functioning, we hypothesized that graduates of the program would show an overall improvement in therapeutic outcomes compared to pre-treatment. Further, we hypothesized that problematic family functioning will be associated with treatment non-responsiveness, and here we present the full program results.

## Materials and methods

### Ethics approval

This work comprises a second-level independent analysis of previously collected, and fully anonymized, clinical data. The data was collected to inform clinical programming and therapeutic approaches. It was secondarily made available to the University of Alberta Department of Psychiatry to conduct an evaluative analysis. This research was approved by the University of Alberta Human Research Ethics Committee (Ethics review number: Pro00089614).

### Study design

The primary aim of this study was to use a secondary analysis of previously collected clinical data to assess the effectiveness of a multimodal intensive treatment program designed for youth survivors of CSA. Using self-report outcome measures collected to inform clinical programming and approach, pre-post treatment data were used to evaluate overall response to treatment for all individuals who completed the full one-year treatment program at the beginning of and immediately after the 1-year treatment program. Secondarily, we aimed to conduct an exploratory descriptive analysis comparing histories of Adverse Childhood Experiences (ACE), resilience, and family functioning to treatment response.

### Participants

All participants in this study were clients at an intensive multimodal treatment program. Admission into the program is determined through a referral and screening process to determine eligibility. Screening is conducted by social workers known as “Transition Coordinators” who work with involved caregivers to determine eligibility. To be considered for the program, the individual must meet the following criteria:

- Be under the age of 18 at the time of admission
- Have experienced at least one incident of sexual abuse that has been reported to a caregiver or trusted adult
- At least one family member or caregiver is identified and remains involved through communication with dedicated Transition Coordinators
- Be psychologically minded and able to benefit from therapy. This is determined through initial screening processes and further determined during the first treatment round, which is considered an assessment round.
  - **Note**: The program aims to accept as many clients as possible. Exclusion generally occurs when there are concerns of violence, active addictions, cognitive functioning that may prevent the participant from benefiting from therapy, or logistical constraints (e.g., travel). The vast majority of screened clients are accepted into the program. Those who are not accepted are often offered entrance into the program at a later date, contingent on stabilization or appropriateness.
- Be medically stable and compliant with prescribed medications
- Have an approximate IQ > 80 as determined through clinical assessment.
  - **Note**: this is not formally assessed unless the individual has received a previous psychoeducational assessment. Cognitive capacity to engage in the program is determined through screening discussions with Transition Coordinator and primary caregivers. Further evaluation occurs during the first round of treatment.

### Completion of a readiness assessment/screening interview

Failure to meet these criteria precludes admission into the treatment program. For the purposes of this study, all included participants were also required to have successfully completed the full one-year treatment program to be included in the analysis.

### Data collection procedure

Data in this study were collected from 01/01/2022–01/01/2026, through the Greenspace Measurement-Based Care (MBC) platform, which abides by all Canadian federal and provincial legislation on privacy and safety of data collection. The Greenspace platform provides built-in validated outcome measures that can be administered electronically via electronic tablets. Clients completed outcome measures at the beginning and end of each of their four treatment episodes. Data collection was facilitated by on-site Child and Youth Workers who provide care for clients when they are not engaged in clinical programming. Data collected on the Greenspace platform was directly converted into a downloadable Excel file for data analysis.

### Treatment facility and program design

The Little Warriors Be Brave Ranch (BBR) is an episodic treatment facility dedicated to treating youth survivors of CSA. Little Warriors is a charitable, not-for-profit organization that aims at prevention, treatment, and education on the impacts of CSA on youth and their families. The BBR is located in a semi-rural area outside of Edmonton, Alberta, Canada. The facility consists of several communal cabins where clients stay during their treatment. The site contains a number of advanced security features to provide safety for children and staff, including a secured fence, a confidential location, security cameras, and 24-hour security at a guarded gate. The facility has been intentionally designed to exceed all required safety and regulatory guidelines and is accredited and licenced by all appropriate authorities. The safety precautions are primarily put in place to ensure that the clients feel safe and secure while on site. The primary aim of the program is to regulate client nervous systems to feel secure and away from danger. There are never any concerns of offenders coming on site; however, there are situations where clients may be in proximity to their offenders in their community. In these cases, dedicated transition coordinators and therapists work with involved caregivers to ensure safety plans are put in place and any concerns are reported to proper authorities.

The BBR provides two separate programs: (1) The Child Program – designed for clients aged 8–12; (2) The Adolescent Program – designed for clients aged 13–17. Both programs consist of four intensive treatment rounds requiring the client to stay on site for the duration of the treatment round. All treatment rounds are 12 days in length, with the exception of the first round of the Child Program, which is 28 days in length. The first round is considered an “assessment” round and is used to evaluate appropriateness for the rest of the program. The extended stay for child participants allows for longer assessment times. Given the social and education factors inherent to adolescents, a 28-day assessment round was not deemed feasible. Each treatment round is separated by approximately three months, which results in a 12-month treatment program. Clients are considered “graduated” upon completion of all four rounds of treatment.

The BBR is a trauma-informed program that both follows the Four Stories conceptual model for treating trauma [32] and is also informed by the framework which integrates the Neurosequential Model of Therapeutics (NMT) [33]. The NMT approach directs the therapeutic approach to focus on anatomically lower brain regions (e.g., brainstem, medulla oblongata) first in order to establish nervous system regulation and attunement. These interventions focus on regulation prior to relationship building and more advanced psychological work such as trauma-focused CBT. The Four Stories approach considers the trauma histories of both the child and their caregiver to ensure that the child-caregiver dyad is in a future directed orientation that optimizes posttraumatic growth.

The intensive program design includes weekly exposure to multiple therapeutic modalities (hence ‘multimodal’), including:

1. Trauma-Focused Cognitive Behavioural Therapy (TF-CBT)
2. Group Therapy
3. Art and Expressive Therapies
4. Music Therapy
5. Animal-Assisted Therapy
6. Individual Psychotherapy
7. Recreational/Play Therapy
8. Cultural Linkages and Activities
9. Life Skills Management Techniques
10. Journaling and Mindfulness Activities

During their time on site, clients also engage in non-programming activities such as mindful movies, recreational games, team building activities, and nature walks. The BBR is set in a camp-like setting in a rural location to minimize any feelings of being in an institution. Although these non-programming activities are not specifically designed to be therapeutic, they contribute to the overall therapeutic milieu and are considered as an essential component of the multimodal program.

Clinical programming is delivered by trained psychologists and clinical social workers who compose the therapeutic team. These modalities are distributed throughout a general schedule that varies depending on the program and the treatment round. The program is specifically designed to provide some variability that can tailor program design to best benefit the needs of the individual client. Informed by the NMT framework, early treatment rounds focus more on nervous system attunement, co-regulation, and modalities that target the development of primal neural networks such as art, animal-assisted, play, and music therapy. Sensory profiles are established to provide the client and caregiver(s) with somatosensory stimulation that can further support in co-regulation. The program also utilizes cultural and healing practices (e.g., religious activities, drumming, sweat-lodge participation, smudging, etc.) to accommodate individuals from diverse cultural backgrounds. Later treatment rounds provide more in-depth TF-CBT strategies, exposure therapeutic techniques, and intensive individual psychotherapy.

Treatment is primarily group-based, with clients being put together into cohorts of 3–8 individuals who complete each treatment round together. Group therapy is incorporated nearly every weekday and includes some component of TF-CBT or psychoeducation. The program design follows these four primary components: (1) Skill-building phase aimed at improving affective, cognitive, behavioural, and biological self-regulation; (2) Gradual exposure to the client’s trauma history in a safe and trauma-informed manner; (3) Cognitive processing of the child’s individual trauma history through the construction of a trauma narrative; (4) Integration of the caregiver(s) and/or family into the treatment process by providing resources and supports. This final component integrates the Four Stories conceptual model that emphasizes the importance of considering the past trauma histories of caregivers and their children together. For the best outcomes to be established, both child and caregiver should be in a present-focused posttraumatic growth orientation [32].

### Outcome measures

#### Posttraumatic stress disorder.

The primary outcome measure for this evaluation was Child PTSD Symptom Scale (CPSS), a validated self-report outcome measure for youth participants and is internally consistent (α = .93) [34]. The CPSS is a 27-item measures that evaluates posttraumatic symptom severity in children and adolescents. Scores range from 0–80, with higher scores indicating a greater severity of PTSD symptoms. A score greater than 30 is considered the clinical threshold for problematic PTSD symptomatology [35].

#### Depression and anxiety.

For participants in the child program, depression and anxiety were measured by the Revised Child Anxiety and Depression Scale (RCADS-25), a 25-item survey that has been validated as a self-report measure for children aged 4–17 [36]. The RCADS-25 has a Cronbach alpha of α = 0.82 [37]. 10 items are dedicated to symptoms of depression, while 3 items are dedicated to assessing levels of Generalized Anxiety Disorder (GAD), Panic Disorder, Social Anxiety Disorder, Social Phobia, and Obsessive-Compulsive Disorder (OCD). Scores are converted into a standardized t-score based on the respondents age and biological sex. A t-score above 70 indicates the clinical threshold for treatment referral [38].

For participants in the adolescent program, depression symptoms were assessed using the Patient Health Questionnaire-adolescent version (PHQ-A). The PHQ-A is a validated self-report measure for depression in adolescents, with an internal consistency of α = 0.82 [39]. The PHQ-A consist of 9-items assessing the severity and frequency of depressive symptoms over the preceding two weeks. Scores range from 0–27, with higher scores indicating a greater level of depressive symptoms. Scores between 15–19 are considered moderately severe, and scores above 20 are considered severe. The PHQ-A was specifically used for the adolescent participants because it includes an item measuring the frequency of suicidal ideation. Anxiety symptoms were measured using the validated 7-item Generalized Anxiety Disorder (GAD-7) scale, with an internal consistency of α = 0.92 [40]. Scores range from 0–21, with higher scores indicate greater levels of anxiety symptoms, scores between 10–15 are considered moderately severe, and scores above 15 are considered severe [41].

#### Quality of life.

Quality of Life was measured using the World Health Organization (WHO) KIDSCREEN-10, a validated and internally consistent (α = 0.82), self-report measure designed for youth [42]. KIDSCREEN-10 consists of 10 items relating to the frequency of positive experiences. Scores range from 0–44, with higher scores indicating improved quality of life.

#### Self-esteem.

Self-esteem was measured using the Rosenberg Self-Esteem Scale (RSES), a validated self-report measure for youth [43].The RSES is a 10 item questionnaire that asks the respondent to report their level of agreement with statements relating to their self-worth and self-esteem. Scores range from 0–30, with higher scores indicating a higher level of self-esteem. Scores below 15 are considered low self-esteem [44]. (Rosenberg, 1965). The RSES has a Cronbach alpha of α = 0.86 [45].

#### Family functioning.

Family functioning was measured using the McMaster Family Assessment Device (FAD), a validated measure to assess family functioning [46]. The questionnaire asks the participant to identify how well each statement relating to family dynamics accurately describes them. Scores range from 12–48, with higher scores indicating higher levels of family dysfunction. The McMaster FAD has reported a Cronbach alpha of α = 0.97 [47].

#### ACEs.

History of childhood adversity was measured using the Center for Youth Wellness Adverse Childhood Experiences Questionnaire (CYW ACE-Q), appropriate for use in with children and adolescents [48]. This survey asks the participant about the original 10 ACE items, with an additional 9 questions pertaining to experiences of bullying, discrimination, witnessing violence, and partner abuse, among others. Scores range from 0–19, with higher scores indicating more ACEs. ACE data was used to provide context of the trauma history of the sample population. Further, this data was used to identify whether treatment non-responsiveness was associated with ACE scores.

#### Resilience.

Resilience was measured using the Child & Youth Resilience Measure (CYRM-R) a validated self-report measure for resilience in youth [49]. The CYRM-R consists of 17 items asking about different markers of resilience apply to the participant and has an internal consistency of α = 0.88 [50]. Scores range from 17–85, with higher scores indicating better resilience and lower scores indicating fewer resilience factors [51]. CYRM-R data was used to identify whether treatment responsiveness was associated with resilience scores.

#### Substance use.

Substance use behaviour was measured using the CRAFFT questionnaire [52]. This measure asks the participant 6 questions relating to using substances in a Car, to Relax, Alone, to Forget, with Friends, or getting into Trouble because of substance use. Scores range from 0−6, with scores above 2 indicating a high risk level for substance abuse [53]. The CRAFFT has a measured Cronbach alpha ranging from α = 0.65–0.86 [54].

### Statistical analysis

Changes in outcome measures were evaluated using paired t-tests to determine statistical significance. The null hypothesis assumed no change in mean outcome measure scores from admission to graduation. To evaluate treatment impact on PTSD, depression, anxiety, self-esteem, substance use, and quality of life, effect sizes were calculated using Cohen’s d. Cohen’s d from 0.2–0.5 was considered a low effect, 0.5–0.8 moderate, and above 0.8 large effect size [55]. An alpha-level of p < 0.05 was used for statistical significance. To correct for false discovery rate, a Benjamini-Hochberg correction was applied to the statistical results. Reductions in overall scores of PTSD, depression, anxiety, family dysfunction, substance abuse, and suicidality were considered clinical improvements. Increases in overall scores of quality of life, self-esteem, and resilience were considered clinical improvements.

Secondarily, response to treatment was assessed by clinically significant changes in reported levels of PTSD. Using the clinical cut-off score of the CPSS, responders were considered individuals who reported PTSD symptoms above the clinical threshold at initial intake, followed by a subclinical score at graduation. Conversely, non-responders were considered individuals who reported PTSD symptoms above the clinical threshold at both intake and graduation. Responders and non-responders were descriptively compared on their histories of trauma using the ACE questionnaire, resilience using the CYRM-R, and family functioning using the McMaster Family Functioning device. The latter measure intended to evaluate the integration of the Four Stories therapeutic model into this program.

### Measurement-based care

The BBR facility implemented a Measurement-Based Care (MBC) approach, which utilizes the routine collection of standardized outcome measures to evaluate and inform programming to maximize treatment benefits [56]. Using the Greenspace MBC platform, clinicians were able to track reported symptoms of their clients to inform their clinical approach.

## Results

### Participant data

In total, 253 participants were admitted into the program, of which 201 (79.4%) participants completed the treatment and were included in the data analysis. Reasons for incompletion include voluntary withdrawal from the program (n = 41, 78.8%), discharge for inappropriate behaviour (n = 6, 11.5%), or clinically assessed as unable to benefit from therapy at present (n = 5, 9.7%). Of the 201 graduates of the program, 85 (42.3%) were from the Child Program and 116 (57.7%) were from the Adolescent Program. A power analysis was conducted to determine adequate sample size for this study. Using paired t-tests with a statistical significance of α = 0.05, statistical power of 0.9, and a measured effect size of 0.5, the minimum sample size was calculated at n = 44.

Demographic data are summarized in **Table 1**. In total, the mean age at initial admission was 13.3 ± 2.4 year. The vast majority of participants were biologically female (n = 166, 82.6%), and 72.6% (n = 146) reported a female gender identity. Twenty-two (10.9%) of participants identified as non-binary. Over a third (n = 72, 35.8%) of participants identified as Indigenous Canadian. Twenty-three (27.1%) of child participants and 27 (23.3%) of adolescent participants were clinically diagnosed with PTSD. Over half of the participants (n = 112, 55.7%) had been prescribed psychotropic medications prior to admission.

**Table 1:** Demographic data for youth CSA survivors completing an intensive multimodal treatment program.

Demographics	Child Program (n = 85)	Adolescent Program (n = 116)	Total (n = 201)
**Mean Age at Intake (years)**	11.5 (1.5)	14.9 (1.6)	**13.3 (2.4)**
**Sex at birth**			
Male	26 (30.6%)	9 (8.0%)	**35 (17.4%)**
Female	59 (69.4%)	103 (92.0%)	**166 (82.6%)**
**Gender Identity**			
Male	25 (29.4%)	8 (6.9%)	**33 (16.5%)**
Female	55 (64.7%)	91 (78.4%)	**146 (72.6%)**
Non-Binary	5 (5.9%)	17 (14.7%)	**22 (10.9%)**
**Indigenous**	30 (35.3%)	39 (34.8%)	**42 (36.2%)**
**Primary Caregiver**			
Biological Parents (together)	16 (18.8%)	25 (21.6%)	**41 (20.4%)**
Biological Mother only	40 (47.1%)	53 (45.7%)	**93 (46.3%)**
Biological Father only	3 (3.5%)	10 (8.6%)	**13 (6.5%)**
CFS	13 (15.3%)	12 (10.3%)	**25 (12.4%)**
Split Custody	4 (4.7%)	4 (3.4%)	**8 (4.0%)**
Relative (e.g., grandparent, aunt/uncle)	8 (9.4%)	9 (7.8%)	**17 (8.5%)**
Adoptive Parents	1 (1.2%)	4 (3.4%)	**5 (2.5%)**
**Diagnosis**			
Anxiety	10 (11.8%)	37 (31.9%)	**47 (23.4%)**
PTSD	23 (27.1%)	27 (23.3%)	**50 (24.9%)**
ADHD	45 (52.9%)	39 (33.6%)	**83 (41.3%)**
Depression	18 (21.2%)	32 (27.6%)	**50 (24.9%)**
FASD	8 (9.4%)	5 (4.3%)	**13 (6.5%)**
ASD	3 (3.5%)	4 (3.4%)	**7 (3.5%)**
BPD	0 (0.0%)	4 (3.4%)	**4 (2.0%)**
ODD	7 (8.2%)	3 (2.6%)	**10 (5.0%)**
Other (e.g., eating disorder, tic disorder)	3 (3.5%)	3 (2.6%)	**6 (3.0%)**
**Medications**			
Antidepressants (e.g., Fluoxetine, Sertraline, Escitalopram)	15 (17.6%)	43 (37.1%)	**58 (28.9%)**
Stimulants (e.g., Methylphenidate, Lisdexamfetamine)	43 (50.6%)	29 (25.0%)	**72 (35.8%)**
Antipsychotics (e.g., Aripiprazole, Quetiapine, Risperidone)	18 (21.2%)	16 (13.8%)	**34 (16.9%)**
Anxiolytics (e.g., Lorazepam, Trazadone)	1 (1.2%)	9 (7.8%)	**10 (5.0%)**

### Clinical outcome data

**Fig 1:**
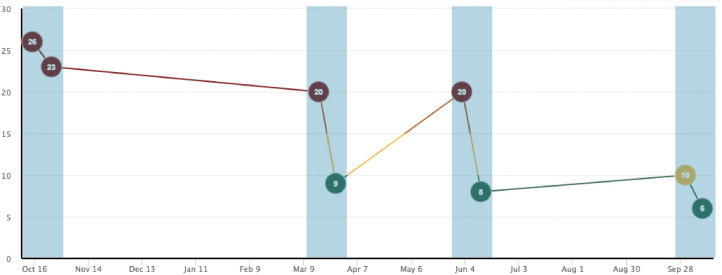
Change in PHQ-A (depression measure) scores over the course of treatment for an adolescent participant. Blue areas represent time when the client was engaged in treatment. Red circles denote severe depression, yellow moderate depression, and green low depression.

**Fig 1** shows a graphical representation of the change in depression symptoms over time, as measured by the PHQ-A. Red circles indicate severe symptoms, yellow moderate symptoms, and green subclinical symptoms. The blue bars denote treatment episodes when the participant was on site. Measures are taken at the beginning and end of each treatment round to provide a detailed representation of treatment effects and change in symptoms over time.

**Fig 2:**
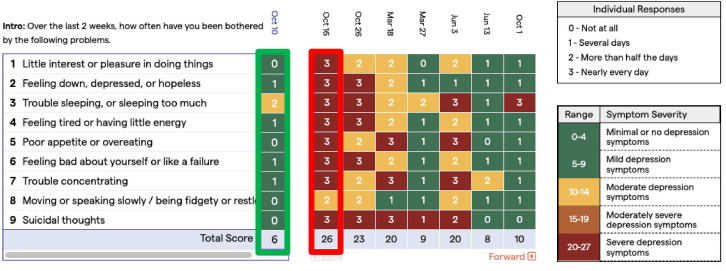
Depression “heat map” representing severity of PHQ-A items over the course of treatment. Red squares denote frequent/severe symptoms, yellow moderate symptoms, and green infrequent/mild symptoms. Treatment progression begins at initial admission (highlighted in red) and moves left to right. The final graduating scores is shown in the far left column (highlighted in green).

**Fig 2** shows a visual “heat map” that depicts the change in severity of specific items on the scale over time. Red represents higher levels of symptoms, orange moderate levels, and green low levels. Symptom scores highlighted in red indicate baseline depression levels. Moving from right to left, the heat map indicates changes in each item over the course of treatment, with each column representing outcome scores at a particular timepoint. The scores highlighted in green on the very left indicate scores at final graduation. The heat map provides a visual representation in the change of symptom severity for each item over the course of time.

**Fig 3:**
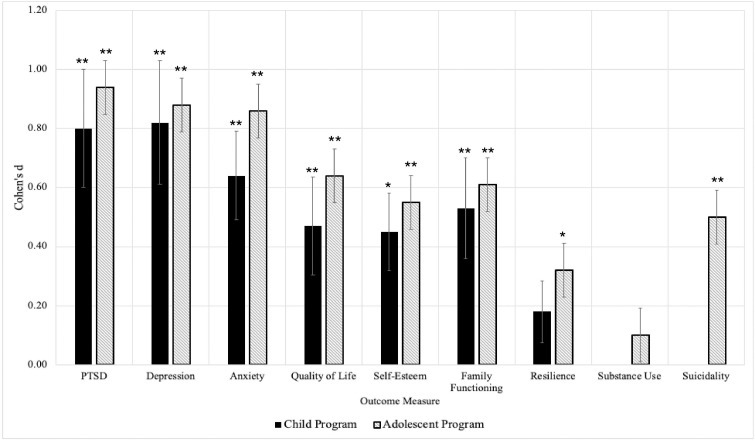
Effect sizes improvements in mental health and quality of life outcomes for child and adolescent graduates of a multimodal treatment program designed for survivors of Child Sexual Abuse (CSA). Effect sizes above 0.8 are considered high, 0.5-0.8 moderate, and below 0.5 low. * denotes p-value <0.05; ** denotes p-value <0.001. **Note:** Substance Use and Suicidality were not collected for child program participants.

Overall clinical outcome data are summarized in **Fig 3**. Paired t-tests showed statistically positive improvements in all measured clinical outcome. Effect sizes ranged from 0.18–0.94. All tests met statistical significance except for changes in substance use in the adolescent program. Note, all p-values represent the adjusted values after applying the Benjamini-Hochberg False Discovery Rate correction.

### Child program results

Child participants (n = 85) reported highly significant positive changes in all measured outcomes when compared from initial admission to graduation from the program (see Table 2). All domains showed positive improvements (i.e., reductions in reported symptoms) and moderate to large effect sizes. The largest change was reported in the PTSD symptoms (Δ% = −35.7%, d = 0.72, p < 0.001), followed by depression (Δ% = −25.3%, d = 0.82, p < 0.001), anxiety (Δ% = −22.5%, d = 0.64, p < 0.001), and reductions in family dysfunction (Δ% = −11.0%, d = 0.53, p < 0.001). Moderate effect sizes were measured for quality of life (d = 0.46, p < 0.001), and self-esteem (0.45, p < 0.001). Resilience scores showed improvement but the effect was small (d = 0.18, p = 0.03).

**Table 2:** Clinical outcome results from child participants (aged 8-12) completing a multimodal treatment program designed for CSA survivors (n = 85).

Outcome Measure	Mean Baseline	Mean Graduation	% Change	Cohen’s d	p-value
PTSD	34.1 (17.5)	21.9 (16.1)	−35.7%	0.72	<0.001
Depression	66.9 (22.6)	50.0 (18.4)	−25.3%	0.82	<0.001
Anxiety	63.6 (23.8)	49.2 (20.8)	−22.5%	0.64	<0.001
Family Dysfunction	28.0 (5.6)	24.9 (6.1)	−11.0%	0.53	<0.001
Quality of Life	26.8 (9.6)	31.3 (9.4)	16.8%	0.47	<0.001
Self-Esteem	16.1 (6.1)	18.8 (5.8)	16.6%	0.45	0.002
Resilience	62.9 (11.0)	65.0 (11.8)	3.4%	0.18	0.03

### Adolescent program results

Adolescent participants (n = 116) reported highly significant positive changes in all measured outcomes when compared to initial admission to graduation from the program, apart from substance use (**Table 3**),. The largest change was reported in PTSD symptoms (Δ% = −38.9%, d = 0.94, p < 0.001), depression (Δ% = −35.0%, d = 0.88, p < 0.001), anxiety (Δ% = 35.6%, d = 0.86, p < 0.001), and reductions in family dysfunction (Δ% = −14.1%, d = 0.61, p < 0.001). All domains showed reductions in reported symptoms and moderate to large effect sizes. Reported suicidal ideation decreased by 44.4% (d = 0.50, p < 0.001). Moderate effect sizes were measured for quality of life (d = 0.64, p < 0.001) and self-esteem (d = 0.55, p < 0.001). Resilience scores showed a highly statistically significant improvement (d = 0.32, p = 0.001), although the effect was relatively small. Substance use scores decreased 12.0% over the course of treatment, but the results did not reach statistical significance for this measure (d = 0.10, p = 0.24).

**Table 3:** Clinical outcome results from adolescent participants (aged 13-17) completing a multimodal treatment program designed for CSA survivors (n = 116).

Outcome Measure	Baseline	Graduation	% Change	Cohen’s d	p-value
PTSD	40.3 (16.9)	24.6 (17.1)	−38.9%	0.94	<0.001
Depression	15.1 (6.1)	9.8 (6.2)	−35.0%	0.88	<0.001
Suicidality	1.1 (1.0)	0.64 (0.9)	−44.4%	0.50	<0.001
Anxiety	12.2 (4.8)	8.1 (5.0)	−35.6%	0.86	<0.001
Substance Use	2.1 (2.1)	1.8 (2.0)	−12.0%	0.10	0.24
Family Dysfunction	29.5 (5.8)	25.3 (7.8)	−14.1%	0.61	<0.001
Quality of Life	24.2 (8.2)	29.1 (7.8)	20.4%	0.64	<0.001
Self-Esteem	13.8 (5.2)	16.8 (5.7)	21.9%	0.55	<0.001
Resilience	59.7 (9.9)	62.8 (11.8)	5.2%	0.32	0.001

### PTSD outcomes

Recovery rate is calculated by the number of individuals who scored above the CPSS clinical cut off for PTSD at baseline, and subsequently scored below this cut off at graduation. Recovery rate for PTSD outcomes is listed in **Table 4**. In the child program, 51 (60.0%) of participants reported a CPSS score above the clinical threshold at admission. This declined to 23 (27.1%) at graduation, a 53.0% recovery rate. In the adolescent program, 80 (71.4%) of participants reported a CPSS score above the clinical threshold at admission. This declined to 32 (28.6%) at graduation, a 60.0% recovery rate.

**Table 4:** PTSD outcomes for child and adolescent graduates of a multimodal treatment program designed for CSA survivors (n = 116).

	Child Program			Adolescent Program		
	**Intake**	**Graduation**	**Percent Change**	**Intake**	**Graduation**	**Percent Change**
Above CPSS threshold	51 (60.0%)	23 (27.1%)	**53.0%**	80 (71.4%)	32 (28.6%)	**60.0%**
**Non-Responders (n = 23)**				**Non-Responders (n = 32)**		
Family Dysfunction	29.3 (5.8)	29.5 (5.3)	**0.7%**	30.9 (7.0)	28.4 (7.7)	**−8.1%**
Resilience	59.6 (11.9)	60 (11.1)	**0.7%**	56.5 (10.1)	59.4 (12.3)	**5.1%**
ACEs	9.1 (3.9)	10.5 (4.5)	**15.4%**	10.7 (3.4)	12.2 (3.4)	**14.0%**
**Responders (n = 62)**				**Responders (n = 80)**		
Family Dysfunction	27.5 (5.4)	23.2 (5.5)	**−15.6%**	28.9 (5.1)	24.0 (7.4)	**−17.0%**
Resilience	64.1 (10.4)	66.8 (11.4)	**4.2%**	61.3 (9.5)	65.1 (11.2)	**6.2%**
ACEs	7.2 (3.3)	8.0 (3.4)	**11.1%**	9.6 (3.2)	10.3 (3.7)	**7.3%**

**Note**: Non-responders are defined as participants who scored above the CPSS clinical threshold for PTSD at baseline and at graduation. Responders are defined as participants who scored below the threshold at graduation.

**Table 4** also shows participants as *Responders* or *Non-Responders*. Responders were any participant whose graduation CPSS score was below the clinical threshold score. This includes participants who reported CPSS scores both above and below the clinical threshold at initial admission. Non-Responders were participants whose graduation CPSS score remained above the clinical threshold. The average change in family dysfunction, resilience, and ACE scores for each group is provided.

Overall, in both the child and adolescent programs, Non-Responders had higher levels of family dysfunction at baseline and graduation. Child Non-Responders reported a slight increase in family dysfunction, while adolescent family dysfunction at graduation (28.4 ± 7.7) was nearly the same as responders family dysfunction at baseline (28.9 ± 5.1). Non-Responders had lower resilience scores at baseline in both programs as compared to Responders. Likewise, Non-Responders had higher ACE scores at baseline and reported a larger change in ACE scores from graduation to baseline.

## Discussion

### Effectiveness of treatment

The findings of this study support our primary hypothesis that a trauma-informed, intensive, and multimodal 1-year treatment program specifically designed to treat youth CSA survivors would demonstrate widespread improvements in multiple dimensions of mental health and well-being. These findings provide preliminary support for this treatment approach in providing benefits to trauma-related symptoms and concurrent afflictions. Given the heterogeneous presentation of CSA effects in survivors, it is reasonable to believe that a multimodal approach would provide the widest benefit to individuals, and research on multimodal therapy has previously been shown to be effective in this context [57], with evidence supporting combinations of TF-CBT and somatosensory therapies (e.g., mindfulness, expressive therapies, play therapy, etc.) for children who have experienced trauma [58]. The present multimodal program utilized a host of modalities to provide access to varied therapeutic approaches that gives the optimal benefit to survivors. The results of this study indicate that this multimodal approach may provide benefit which extend beyond PTSD-specific symptoms, including enhancement of mood, bolstering of self-esteem, increased life satisfaction, improved family functioning, and reductions in suicidal ideation. This last point is particularly critical given the high rates of self-harm and suicidal behaviour that is common in survivors of early-life trauma [59].

Supporting the use of the NMT-informed framework [33] for this population, the findings suggest that prioritizing nervous system attunement, prior to intensive psychotherapy or trauma-focused interventions, appeared to be an effective approach for trauma-focused treatment. To this end, the program specifically adapts the therapeutic milieu to provide a consistent sense of safety for the participants. Although the multimodal approach to treatment is important, it is essential to consider the setting that treatment takes place in, and how this can be integrated into an NMT approach. The camp-like setting of the BBR removes the traditional “institutional” feel that can increase stigma to treatment and make a child or adolescent feel “sick” or “broken”. Trauma Systems Therapy (TST) approaches emphasize the importance of controlling for the environment to promote a sense of safety whenever possible [60]. Having a balance between clinical programming and recreational activities (e.g., watching movies, playing games, spending time outdoors, etc.) can prevent therapeutic burnout and keep youth engaged in the treatment process. Although the multimodal design provides diverse therapeutic options to holistically treat the child or adolescent, some participants may have been able to attain similar outcomes with less intensive treatment such as outpatient or remote therapy. The results of this study suggest that less intensive treatment options that prioritize trauma-informed programming, with an emphasis on establishing a safe environment to reattune dysregulated nervous system activation, are safe, appropriate, and beneficial for this population.

Additionally, we are aware that the lack of an active control population limits our ability to determine the level of spontaneous improvement in untreated patients, as it would be expected that at least some of the individuals would experience this. However, there is little historical evidence that is directly applicable to this, particularly given the long-term longitudinal nature of this program. This issue is discussed more in the limitations section, but the longitudinal design of the program deserves specific consideration as well. As evidenced in **Figs 1** and **2**, many of the positive gains that occur during treatment may not be sustained if the youth is to return to an environment which does not facilitate the maintenance of the strategies developed in treatment. The episodic approach allows for the child to undergo an intensive treatment round to learn these strategies, followed by a “training period” where they can implement these skills in a real-world setting. With each subsequent round, the client is able to iteratively hone these strategies through corrective updating. Further, a longitudinal approach can minimize the impact of spontaneous improvement which may occur outside the context of treatment. This further emphasizes the importance of caregiver involvement in the treatment process and the impact caregiver buy-in has on the maintenance of these improvements [61].

### Four Stories and caregiver considerations

The BBR utilizes the Four Stories conceptual framework which outlines the importance of incorporating caregiver trauma history and mental orientation when treating children and youth with trauma histories. By providing caregivers with parental resources, psychoeducational programs, and family supports, the goal is to empower caregivers to become long-term facilitators of the skills and strategies the youth develops during their time in treatment. The findings of this study support the utilization of the Four Stories framework in moving children and families through trauma. In the overall sample, reported family functioning improved for both children and adolescent participants. Further, individuals who were less responsive to treatment were more likely to have high levels of family dysfunction compared to participants who reported clinical improvements in trauma symptoms. Evidence has shown that integration of caregivers and families into trauma treatment is beneficial [62]. Parental beliefs about trauma may be misinformed which may hinder treatment outcomes. In a trauma context, it is crucial to consider a caregiver’s personal history and perspective on trauma as this can have a significant influence on their beliefs and emotions towards their child and themselves [31]. The Four Stories approach aims at concurrently directing caregiver and child into a present-focused posttraumatic growth orientation to maximize treatment outcomes [32].

In this study, reported family dysfunction reduced in both child and adolescent participants, providing evidence that family functioning and treatment outcomes are intertwined. Based on this model, we hypothesized that decreased family functioning will be associated with treatment non-responsiveness and poorer outcomes. When differentiating participants between *Responders* and *Non-Responders* in terms of PTSD symptoms, we found that those participants whose PTSD symptoms remained above the clinical threshold score were more likely to report dysfunctional family dynamics at baseline. Further, those participants had significantly less improvement in family functioning, with Non-Responders in the child program reporting worse family functioning over the course of treatment. Adolescent participants who did respond to treatment showed the greatest change in family functioning, providing further support that family functioning and treatment outcomes are associated. It should be noted that over 10% of the participants’ legal guardians were CFS workers, suggesting that these clients were from foster or group homes. Depending on the circumstances, it can be difficult to fully integrate caregiver work into the treatment approach and that may warrant specific considerations on how to best support these individuals. Non-Responders were also more likely to have lower resilience scores at admission and graduation, more complex and severe histories of trauma, and a higher likelihood of re-traumatization, as evinced by the changes in CYRM-R and CYW ACE-Q scores. Trauma histories are associated with treatment non-responsiveness; this is particularly true for individuals with complex trauma, which may have been more intensive, prolonged, or violent, or individuals who experience ongoing traumatic experiences concurrent with treatment [63]. As such, it is unsurprising that Non-Responders were more likely to have higher ACE scores and more complex trauma histories. Markers for resilience, including enhanced family support, access to resources, clear goals for achievement, and inclusion into social networks were much lower for non-responders, which suggests that facilitating these factors may optimize treatment outcomes. Treatment approaches that consider resilience factors prior to treatment and aim to bolster these factors when possible may provide further benefit to this population.

One interesting finding of this study was that reported substance use did not show statistically significant change and did increase in a number of adolescent participants. Given that the mean age of adolescent participants at admission was roughly 13 years old, it is not surprising that substance use increased as adolescents, especially those with trauma histories, generally begin engaging in unsafe behaviours between the ages of 13–16 [64]. There is also the possibility that reporting became more transparent as clients became more comfortable completing the surveys and being in the program over time.

### Limitations of the study

There are several potential limitations to consider in this study which may impact the generalizability of the findings. First, this was a single-arm pre/post research design, and there was no direct control group to compare outcomes. Collecting outcome measures in an intensive child and youth treatment center can be challenging, and finding an adequate comparison group is problematic. While past studies have used a waitlist control model, with a 1-year treatment program this approach is not feasible, but we continue to seek approaches to allow a control group to be compared to the program. Importantly, the data collected came from a convenience sample of pre-collected sequential clinical data, which provided the researchers with a unique opportunity to evaluate a novel program and provide preliminary insight into the program’s potential efficacy. Given the relatively large sample size, as well as the findings of the statistical analysis, the researchers believe that the findings of this paper provide evidence of treatment efficacy, despite the absence of a comparison sample. Nonetheless, further evaluation that includes waitlist controls for short-time periods (to compare outcomes at 3-months), or outcomes from a different child and youth treatment center, is necessary to further validate these findings. Supporting the need for future research to also contain control groups is exemplified by recent literature suggesting that untreated CSA can lead to negative long-term outcomes and significant economic strain on social and health programs [65,66]. Without a control group, many of the current findings are not generalizable to the larger population and positive outcomes may be attributed to external factors, such as expectancy effects, natural recovery over time, outside supports or counselling, and regression to the mean. While the large sample size in this study does give support for treatment effects, without appropriate control groups, causality cannot be fully concluded.

Second, this study relied on self-report outcome measures which are subject to bias and interpretation. However, given the sample size and the number of different outcomes measured, the findings have relatively strong power to control for this subjectivity, reflected in the effect size results. Dedicated child and youth workers facilitated data collection to ensure completion by each client while on site. Supplementing these data with outcomes collected from caregivers, therapists, and/or relevant stakeholders may increase the richness of this evaluation. Third, we are unable to control for the non-specific components of the multimodal program, or to determine the relative benefits from this compared to more formal components. Adherence to medications while in treatment is a mandatory requirement of the program. As there was significant variability in adherence to medications when not on site, some of the benefits of therapy may be attributed to pharmacological effects. That being said, advocating for pharmacological treatment based on therapeutic observations is a component of the treatment approach. Finally, the absence of caregiver data prevented a full exploration into the impact of treatment on the caregiver-child dyad. The family functioning measure collected aimed to address the impact of treatment on family functioning, but direct input from caregivers may have further enhanced the findings of this study.

### Future research

Although this manuscript provides support for this treatment approach, further research is needed to optimize the evidence base. Ideally a comparison group or waitlist control could be utilized to compare treatment outcomes and further delineate the effects of multimodal treatment. Future studies could also include outcomes collected from caregivers over the course of treatment to better understand the effect of the Four Stories approach. Using demographic data collected at baseline, a multivariate regression on demographic factors on treatment outcomes may help elucidate who is most likely to benefit from treatment and how to better identify those who may be treatment resistant. This is an intensive program, and is hard to scale up to meet the huge unmet need. Determining which combination of modalities is most effective may help with future scaling to meet this need. Finally, further investigation into the long-term effects of treatment may also need to be considered. Follow-up surveys can provide insight into the sustainability of treatment effects.

## Conclusion

This research study provided evidence that an intensive multimodal approach to treating youth survivors of CSA is safe and appears effective. Given the impact of early-life stress and trauma on nervous system development, adopting an NMT-informed approach which focuses on nervous system attunement prior to intensive therapeutic work may provide the best benefit for this population. The Four Stories approach considers the importance of orienting both child and caregiver towards posttraumatic growth. Integrating caregivers and families into the therapeutic process may be the best approach to ensure that treatment outcomes are maintained throughout the treatment process. Finally, incorporating a trauma-informed and family-based approach to the multimodal treatment of youth CSA survivors may provide the largest benefit to this population, but further investigation into the sustainability of post-treatment outcomes is needed. Further, comparisons to control groups is necessary to establish full treatment impact.
