# Supplementary material for: Evaluation of outcomes in 201 child and youth survivors of Child Sexual Abuse (CSA) who participated in a one-year multimodal treatment program
Source: PLoS One. 2026 Jul 23;21(7):e0354692. doi: 10.1371/journal.pone.0354692 (PMC13395316; doi:10.1371/journal.pone.0354692)
Supplement: Attachment — Submitted filename: Response to Reviewers.docx [file pone.0354692.s002.docx]

**Response to Reviewers**

**Reviewer #1:** This is a relatively large follow-up of a treatment programme for child-and adolescent-PTSD after exposure to sexual abuse. Although I accept the idea that it is meaningful to do this follow-up without a control group I still feel that a discussion of expected spontaneous improvement in untreated patients would have been warranted. In fact, very little improvement could be expected without treatment but that needs to be discussed based on previous literature.

Thank you for your comment. This has been addressed in the discussion portion of the manuscript, with key considerations outlined for the impact of no treatment. We believe the sample size of this manuscript does account for spontaneous improvement that may not be attributable to treatment. This has been noted in the limitations section.

In the description of the programme I lack one piece of information: What about the offenders? They obviously did not participate at the centre, but the reader notes the high security precautions with fences etc. One gets the impression that there may be real dangers around this. And if it is dangerous during those treatment periods it is likely to be dangerous at home as well. I also realise that some of the patients might flee it there are no precautions against that. On the whole, a description of communication with responsible caregivers would help the reader.

Thank you for noting this. The safety precautions are primarily put in place to ensure that the clients feel safe and secure while on site. The primary aim of the program is to regulate client nervous systems to feel secure and away from danger. There are never any concerns of offenders coming on site; however, there are situations where clients may be in proximity to their offenders in their community. In these cases, dedicated transition coordinators work with involved caregivers to ensure safety plans are put in place and any concerns are reported to proper authorities. This has been noted in the manuscript.

In the inclusion/excusion criteria there is one that draws my attention:

"Be psychologically minded and able to benefit from therapy"

This seems like a soft criterion that could enfathom anything including, “I like this person”. I know it is impossible to treat people who are hostile but still, this is also something that should be discussed. In the end it would reveal what proportion of children with these problems that the treatment programme would accept. If possible, one wonders what percentage of considered patients what was accepted.

This has been addressed in the methods section. The vast majority of clients screened are accepted into the program, as the first round of treatment is considered an assessment round and is used to determine further appropriateness for the program. The program aims to treat as many clients as possible and generally only excludes potential participants because of concerns of violence, active addictions, cognitive functioning that may prevent active participation in psychoeducation and therapy, or logistical constraints.

**Reviewer #2:** Thank you for the opportunity to review this manuscript. The authors examined the impact of a one-year multimodal treatment program for survivors of child sexual abuse (CSA), an important area of research. While the topic is meaningful and the study has the potential to contribute to the literature, several issues currently limit the clarity, coherence, and overall contribution of the manuscript.

Overall, the paper would benefit from improved organization and clearer articulation of key decisions. The introduction at times feels unfocused, with some content (e.g., prevalence estimates and Canada-specific context) appearing somewhat disconnected from the study’s aims, while other important areas—such as a more detailed overview of the treatment approaches (particularly the Four Stories framework)—require further development. Streamlining and strengthening the narrative would help situate the study more effectively.

Thank you for your comments and for your suggestions. We have re-worked the introduction to be more coherent and in line with the study aims, including further details on the Four Stories framework.

The most significant concerns relate to the methods section. Important details are either missing or insufficiently described, including clearer justification for the inclusion of specific variables, the role of certain constructs (e.g., ACEs), and how the various therapeutic components fit together conceptually and analytically. Additional information on the measures (e.g., scoring, response options, and psychometric properties) is needed to support interpretability. Greater clarity is also required regarding the statistical analysis plan. In particular, the use of multiple t-tests raises concerns given the likely intercorrelations among outcome variables; alternative approaches (e.g., multivariate analyses) may be more appropriate. Clarification of the analytic strategy, including software used and handling of key variables, would strengthen confidence in the findings. The absence of a control or comparison group should also be more explicitly acknowledged.

Thank you for noting this. The methods section has been updated to include more information of the specific variables and the roles of these constructs in the analysis. Relevant references to the outcome measures used have also been included.

The use of multiple t-tests was accounted for using the Benjamini-Hochberg false discovery rate correction technique. This has been noted in the statistical analysis section. The absence of a comparison group has been explicitly acknowledged and noted in the limitations section.

In the results section, there is some redundancy between the text and tables, and opportunities to streamline presentation (e.g., summarizing rather than repeating table values). Greater clarity in figures and tables (including consistent terminology and clear labeling of acronyms) would improve readability.

Thank you for this comment. We have edited the results section to remove redundancies and make the summary more streamline. Clarify for figures, ables, and acronyms has been adjusted accordingly.

The discussion would benefit from closer alignment with the study’s actual data and analyses. At times, the authors interpret findings or emphasize factors (e.g., treatment setting, caregiver involvement, and the Four Stories framework) that were not directly measured or tested, which weakens the strength of the conclusions. Tightening this section to focus on what can be directly supported by the data would enhance the manuscript’s credibility.

The discussion section has been updated to be more aligned with the findings of the study. Further discussion on the relevance of certain measures to the Four Stories framework has been included.

Finally, there are a number of minor issues related to clarity, grammar, and consistency (e.g., tense – sometimes past, sometimes present, spelling (e.g. behavior/behaviour), and typographical errors) that should be addressed throughout.

Thank you for this, these changes have been addressed in the manuscript.

In summary, this study addresses an important topic and presents potentially valuable findings. However, revisions—particularly to the methods, analytic rationale, and alignment between aims, analyses, and interpretation—are needed to strengthen the manuscript and clarify its contribution to the field.

Thank you for your review and for your insightful comments. We hope that the changes have addressed the reviewers concerns and is stronger going forward.

**Reviewer #3**: This study concerns a development and evaluation of a multimodal treatment program for survivors of child sexual abuse. The article is well-written and concerns an interesting and important topic.

Some remarks

Abstract
Minor: the sentence ”Participants whose PTSD scores remained above the clinical threshold at graduation were had
higher levels of family dysfunction”: contains an extra word ”were had”.

Thank you, this has been adjusted.

Material and methods
• This study contains a "second level independent analysis", which makes one wonder whether there is a 1st level analysis reported elsewhere?

This data has not been reported elsewhere. The data is collected as a part of the program design to inform therapists’ about their clients. The program released this data to the academic institution to conduct a secondary analysis on pre-collected clinical data. This has been clarified in the manuscript.

• The screening of participants was a bit unclear, was this done by psychologists? It could be good to clarify.

Thank you for noting this. Dedicated social workers known as transition coordinators conduct the screening interviews. This has been noted in the manuscript.
• In the limitations, the authors mention that caregiver data is missing. It would have been good to also mention the risk that the accompanying parent was the abuser him/herself, was that prevented in any way?

No offenders are included in the treatment process, regardless of affiliation to the child (e.g., biological parent). This is ensured in the screening process.

Discussion and conclusion
* Here, it would have been good with some mentioning of how the results could perhaps be generalized to other types of less intensive treatments?

Thank you, this has been added to the discussion section.

**Reviewer #4:** This manuscript addresses an important and difficult clinical area, and overall it reads as a solid, potentially valuable paper. The topic is relevant, the program description is detailed, and the authors appear committed to improving care for a highly vulnerable population. At the same time, in its current form the manuscript has several conceptual, methodological, and editorial issues that should be addressed before publication.

My main concern is that the paper at times overstates what can be concluded from the design. This is an uncontrolled pre-post evaluation of a residential program, apparently without preregistration. For a secondary outcome analysis of this kind, the absence of preregistration is an important limitation. More importantly, without a control group, the results cannot be interpreted as evidence of efficacy in a causal sense. Improvements may reflect treatment effects, but they may also reflect passage of time, regression to the mean, expectancy effects, environmental containment, or natural recovery. Therefore, language such as “effective” or claims implying confirmation of efficacy should be substantially softened throughout the manuscript. Relatedly, the statement that a relatively large sample and large effect sizes compensate for the lack of a control group is methodologically incorrect; effect sizes in uncontrolled designs do not establish causality.

Thank you for noting this concern. This has been addressed in the manuscript, with changes to the claims being softened to align with the statistical results and analysis.

The abstract states that “no gold standard currently exists,” yet the Introduction reportedly describes TF-CBT as a gold standard treatment. There is also an error (“were had higher levels”). Finally, the lack of a control group should already be acknowledged in the abstract.

Thank you for pointing out this inconsistency. The language in the manuscript has been adjusted and the lack of control group acknowledged in the abstract.

The Introduction is generally concise and well structured. However, some sections feel overly local or insufficiently integrated for an international readership (discussion of Canadian legal terminology reform and Canadian prevalence data as the only national epidemiological context). The section on AI and CSAM is potentially interesting, but currently reads as underdeveloped and has no references.

Thank you for noting this. The introduction has been adjusted to be more relevant for an international audience and a relevant citation has been added.

The final hypothesis refers to “long-term results,” which is misleading if the program lasts one year and no follow-up data were collected.

This language has been adjusted in the final hypothesis.

The Methods section raises several concerns regarding sampling and internal validity. Inclusion criteria such as being “psychologically minded and able to benefit from therapy” are highly subjective and not operationalized; the manuscript should clarify who assessed this and according to what criteria. Similarly, IQ >80 based on “clinical assessment” is too vague. A more serious issue is that completion of the full program appears to be required for inclusion in the analysis. This creates a classic completion bias: only those who successfully completed treatment are analyzed. Since 52 participants (253 to 201) did not complete the program, the paper should report dropout analyses comparing completers vs non-completers on baseline variables. Without this, results may be substantially biased and this should be explicitly discussed in the limitations.

Thank for you noting these concerns in the methods section. The screening process has been described in more detail to provide context relating to inclusion. In general, the program aims to include as many participants as possible. An estimated 80-90% of screened participants are admitted into the program. However, some participants are not immediately admitted into the program and require medical or psychological stabilization before being accepted. Many individuals who are not immediately accepted into the program eventually end up being accepted when there is more stabilization, or there is a more appropriate cohort for the participant.

Our intention is to publish a follow-up study that compares demographic and baseline characteristics to treatment completion and response, which will include the 52 non-completers that have been excluded for this study.

The intervention itself is described in commendable detail. However, I could not identify whether the program was manualized or whether treatment fidelity was monitored.
Several measurement descriptions also need correction. KIDSCREEN-10 and CRAFFT reportedly contain placeholder citations (“REFS”). CYRM-12 is described as a 17-item measure, while its name suggests 12 items. Reliability coefficients are missing of all measures.

This has been updated into the methods section. Treatment follows a manualized, group-based program. Treatment fidelity is monitored through documentation software known as Salesforce. All references have been updated.

Reliability coefficients have been included for all measures where relevant.

In the Results section, a power analysis is missing. Given subgroup sizes of n=85 and n=116 and the number of tested variables, this should be addressed. More than half of participants (55.7%) received pharmacological treatment, yet no attempt appears to have been made to control for medication effects. Changes in PTSD, depression, or related outcomes may therefore be partly pharmacological rather than attributable to the program alone. Heading of Table 4 reportedly lists n=116 (Adolescent Program) although the table contains data from both programs.

Thank you noting this. A power analysis has been included in the results section.

Medication effects were not measurable as many participants had been taking their meds for a variable amount of time prior to initial intake. There were also differences to participant adherence to medications when not in programming. The impact of potential adherence to medications while in treatment has been noted in the limitations portion of the discussion.

Thank you for noting the error in Table 4. This has been adjusted.

The Discussion would benefit from a more cautious interpretation of findings. At present, the conclusions seem stronger than warranted by the design. This study can reasonably be framed as showing promising pre-post improvements and feasibility in a real-world residential setting, but not definitive treatment efficacy. The limitations section is commendably transparent in acknowledging several key issues (lack of control group, self-report data, absence of caregiver data). However, additional limitations should be included: completion bias, lack of follow-up, possible confounding by medication, absence of manualization/fidelity checks, single-site design, and possible social desirability bias in self-report data collected in a residential context with staff involvement. If I understand correctly, the authors are also evaluating their own program within a single center; if so, potential conflicts of interest or allegiance effects should be transparently disclosed.

The discussion has been adjusted to communicate more cautious interpretations of the findings and the limitations portion has been bolstered to account for these cautions.

The Data Availability Statement is unclear. It is not evident why de-identified data could not be shared, at least through controlled or open access, if they have been properly anonymized. The authors should clarify the specific barriers to data sharing.
In summary, this is a worthwhile manuscript on an important topic, but the current version requires revision.

Thank you for noting this. The collected data is used to inform therapeutic programming and has been granted for secondary analysis through an academic institution. De-identified data is available upon request to the data holder whose contact information is provided in the data availability statement.
