# Supplementary material for: Evaluation of outcomes in 201 child and youth survivors of Child Sexual Abuse (CSA) who participated in a one-year multimodal treatment program
Source: PLoS One. 2026 Jul 23;21(7):e0354692. doi: 10.1371/journal.pone.0354692 (PMC13395316; doi:10.1371/journal.pone.0354692)
Supplement: Peer review history

# Peer review: Decision Letter 0

**DOI:** 10.1371/journal.pone.0354692.r001

**Authors:** Vincenzo De Luca

1 May 2026

PONE-D-26-10574

Development and evaluation of a successful one-year multimodal treatment program for youth survivors of Child Sexual Abuse (CSA)

PLOS One

Dear Dr. Reeson,

Thank you for submitting your manuscript to PLOS ONE. After careful consideration, we feel that it has merit but does not fully meet PLOS ONE’s publication criteria as it currently stands. Therefore, we invite you to submit a revised version of the manuscript that addresses the points raised during the review process.

Please submit your revised manuscript by Jun 15 2026 11:59PM. If you will need more time than this to complete your revisions, please reply to this message or contact the journal office at plosone@plos.org. When you’re ready to submit your revision, log on to https://www.editorialmanager.com/pone/ and select the 'Submissions Needing Revision' folder to locate your manuscript file.

Please include the following items when submitting your revised manuscript:

- A letter that responds to each point raised by the academic editor and reviewer(s). You should upload this letter as a separate file labeled 'Response to Reviewers'.
- A marked-up copy of your manuscript that highlights changes made to the original version. You should upload this as a separate file labeled 'Revised Manuscript with Track Changes'.
- An unmarked version of your revised paper without tracked changes. You should upload this as a separate file labeled 'Manuscript'.

If you would like to make changes to your financial disclosure, please include your updated statement in your cover letter. Guidelines for resubmitting your figure files are available below the reviewer comments at the end of this letter.

If applicable, we recommend that you deposit your laboratory protocols in protocols.io to enhance the reproducibility of your results. Protocols.io assigns your protocol its own identifier (DOI) so that it can be cited independently in the future. For instructions see: https://journals.plos.org/plosone/s/submission-guidelines#loc-laboratory-protocols. Additionally, PLOS ONE offers an option for publishing peer-reviewed Lab Protocol articles, which describe protocols hosted on protocols.io. Read more information on sharing protocols at https://plos.org/protocols?utm_medium=editorial-email&utm_source=authorletters&utm_campaign=protocols.

As the corresponding author, your ORCID iD is verified in the submission system and will appear in the published article. PLOS supports the use of ORCID, and we encourage all coauthors to register for an ORCID iD and use it as well. Please encourage your coauthors to verify their ORCID iD within the submission system before final acceptance, as unverified ORCID iDs will not appear in the published article. *Only*  the individual author can complete the verification step; PLOS staff *cannot*  verify ORCID iDs on behalf of authors.

We look forward to receiving your revised manuscript.

Kind regards,

Vincenzo De Luca

Academic Editor

PLOS One

Journal Requirements:

When submitting your revision, we need you to address these additional requirements.

1. Please ensure that your manuscript meets PLOS ONE's style requirements, including those for file naming. The PLOS ONE style templates can be found at

https://journals.plos.org/plosone/s/file?id=wjVg/PLOSOne_formatting_sample_main_body.pdf and https://journals.plos.org/plosone/s/file?id=ba62/PLOSOne_formatting_sample_title_authors_affiliations.pdf

2. We note that you have indicated that there are restrictions to data sharing for this study. For studies involving human research participant data or other sensitive data, we encourage authors to share de-identified or anonymized data. However, when data cannot be publicly shared for ethical reasons, we allow authors to make their data sets available upon request. For information on unacceptable data access restrictions, please see http://journals.plos.org/plosone/s/data-availability#loc-unacceptable-data-access-restrictions.

Before we proceed with your manuscript, please address the following prompts:

a) If there are ethical or legal restrictions on sharing a de-identified data set, please explain them in detail (e.g., data contain potentially identifying or sensitive patient information, data are owned by a third-party organization, etc.) and who has imposed them (e.g., a Research Ethics Committee or Institutional Review Board, etc.). Please also provide contact information for a data access committee, ethics committee, or other institutional body to which data requests may be sent.

b) If there are no restrictions, please upload the minimal anonymized data set necessary to replicate your study findings to a stable, public repository and provide us with the relevant URLs, DOIs, or accession numbers. Please see http://www.bmj.com/content/340/bmj.c181.long for guidelines on how to de-identify and prepare clinical data for publication. For a list of recommended repositories, please see https://journals.plos.org/plosone/s/recommended-repositories. You also have the option of uploading the data as Supporting Information files, but we would recommend depositing data directly to a data repository if possible.

Please update your Data Availability statement in the submission form accordingly.

3. In the online submission form, you indicated that data cannot be shared publicly because the data analyzed for this study was collected as part of programming at a multimodal treatment center for child sexual abuse (CSA) survivors. This data is collected by the program as a means of informing treatment and clinical approaches. De-identified data were provided for the research team to conduct a secondary analysis on pre-collected data. As such, the original data is not available for open access as it is personal information collected by the treatment center. The researchers do not have consent to share the de-identified data at this time without a specific request from researchers who meet the criteria for access to confidential data. Data are available from the Little Warriors Clinical Director, Melanie Jonkman (Email: Melanie@littlewarriors.ca; Phone: 780-952-4301) for researchers who meet the criteria for access to confidential data

All PLOS journals now require all data underlying the findings described in their manuscript to be freely available to other researchers, either 1. In a public repository, 2. Within the manuscript itself, or 3. Uploaded as supplementary information.

This policy applies to all data except where public deposition would breach compliance with the protocol approved by your research ethics board. If your data cannot be made publicly available for ethical or legal reasons (e.g., public availability would compromise patient privacy), please explain your reasons on resubmission and your exemption request will be escalated for approval.

4. For studies involving third-party data, we encourage authors to share any data specific to their analyses that they can legally distribute. PLOS recognizes, however, that authors may be using third-party data they do not have the rights to share. When third-party data cannot be publicly shared, authors must provide all information necessary for interested researchers to apply to gain access to the data. (https://journals.plos.org/plosone/s/data-availability#loc-acceptable-data-access-restrictions)

For any third-party data that the authors cannot legally distribute, they should include the following information in their Data Availability Statement upon submission:

1) A description of the data set and the third-party source

2) If applicable, verification of permission to use the data set

3) Confirmation of whether the authors received any special privileges in accessing the data that other researchers would not have

4) All necessary contact information others would need to apply to gain access to the data

5. Please provide a complete Data Availability Statement in the submission form, ensuring you include all necessary access information or a reason for why you are unable to make your data freely accessible. If your research concerns only data provided within your submission, please write "All data are in the manuscript and/or supporting information files" as your Data Availability Statement.

6. When completing the data availability statement of the submission form, you indicated that you will make your data available on acceptance. We strongly recommend all authors decide on a data sharing plan before acceptance, as the process can be lengthy and hold up publication timelines. Please note that, though access restrictions are acceptable now, your entire data will need to be made freely accessible if your manuscript is accepted for publication. This policy applies to all data except where public deposition would breach compliance with the protocol approved by your research ethics board. If you are unable to adhere to our open data policy, please kindly revise your statement to explain your reasoning and we will seek the editor's input on an exemption. Please be assured that, once you have provided your new statement, the assessment of your exemption will not hold up the peer review process.

7. Please update your submission to use the PLOS LaTeX template. The template and more information on our requirements for LaTeX submissions can be found at http://journals.plos.org/plosone/s/latex.

8. Please include a separate caption for each figure in your manuscript.

9. We note that Figure 2 in your submission contain copyrighted images. All PLOS content is published under the Creative Commons Attribution License (CC BY 4.0), which means that the manuscript, images, and Supporting Information files will be freely available online, and any third party is permitted to access, download, copy, distribute, and use these materials in any way, even commercially, with proper attribution. For more information, see our copyright guidelines: http://journals.plos.org/plosone/s/licenses-and-copyright.

We require you to either (1) present written permission from the copyright holder to publish these figures specifically under the CC BY 4.0 license, or (2) remove the figures from your submission:

a. You may seek permission from the original copyright holder of Figure 2 to publish the content specifically under the CC BY 4.0 license.

We recommend that you contact the original copyright holder with the Content Permission Form (http://journals.plos.org/plosone/s/file?id=7c09/content-permission-form.pdf) and the following text:

“I request permission for the open-access journal PLOS ONE to publish XXX under the Creative Commons Attribution License (CCAL) CC BY 4.0 (http://creativecommons.org/licenses/by/4.0/). Please be aware that this license allows unrestricted use and distribution, even commercially, by third parties. Please reply and provide explicit written permission to publish XXX under a CC BY license and complete the attached form.”

Please upload the completed Content Permission Form or other proof of granted permissions as an "Other" file with your submission.

In the figure caption of the copyrighted figure, please include the following text: “Reprinted from [ref] under a CC BY license, with permission from [name of publisher], original copyright [original copyright year].”

b. If you are unable to obtain permission from the original copyright holder to publish these figures under the CC BY 4.0 license or if the copyright holder’s requirements are incompatible with the CC BY 4.0 license, please either i) remove the figure or ii) supply a replacement figure that complies with the CC BY 4.0 license. Please check copyright information on all replacement figures and update the figure caption with source information. If applicable, please specify in the figure caption text when a figure is similar but not identical to the original image and is therefore for illustrative purposes only.

10. If the reviewer comments include a recommendation to cite specific previously published works, please review and evaluate these publications to determine whether they are relevant and should be cited. There is no requirement to cite these works unless the editor has indicated otherwise.

[Note: HTML markup is below. Please do not edit.]

Reviewers' comments:

Reviewer's Responses to Questions

**Comments to the Author**

1. Is the manuscript technically sound, and do the data support the conclusions?

The manuscript must describe a technically sound piece of scientific research with data that supports the conclusions. Experiments must have been conducted rigorously, with appropriate controls, replication, and sample sizes. The conclusions must be drawn appropriately based on the data presented.

Reviewer #1: Yes

Reviewer #2: Partly

Reviewer #3: Yes

Reviewer #4: Partly

**********

2. Has the statistical analysis been performed appropriately and rigorously?

Reviewer #1: Yes

Reviewer #2: No

Reviewer #3: Yes

Reviewer #4: Yes

**********

3. Have the authors made all data underlying the findings in their manuscript fully available?

The PLOS Data policy requires authors to make all data underlying the findings described in their manuscript fully available without restriction, with rare exception (please refer to the Data Availability Statement in the manuscript PDF file). The data should be provided as part of the manuscript or its supporting information, or deposited to a public repository. For example, in addition to summary statistics, the data points behind means, medians and variance measures should be available. If there are restrictions on publicly sharing data—e.g. participant privacy or use of data from a third party—those must be specified.

Reviewer #1: No

Reviewer #2: No

Reviewer #3: Yes

Reviewer #4: No

**********

4. Is the manuscript presented in an intelligible fashion and written in standard English?

PLOS ONE does not copyedit accepted manuscripts, so the language in submitted articles must be clear, correct, and unambiguous. Any typographical or grammatical errors should be corrected at revision, so please note any specific errors here.

Reviewer #1: Yes

Reviewer #2: Yes

Reviewer #3: Yes

Reviewer #4: Yes

**********

5. Review Comments to the Author

Please use the space provided to explain your answers to the questions above. You may also include additional comments for the author, including concerns about dual publication, research ethics, or publication ethics. (Please upload your review as an attachment if it exceeds 20,000 characters)

Reviewer #1: This is a relatively large follow-up of a treatment programme for child-and adolescent-PTSD after exposure to sexual abuse.

Although I accept the idea that it is meaningful to do this follow-up without a control group I still feel that a discussion of expected spontaneous improvement in untreated patients would have been warranted. In fact, very little improvement could be expected without treatment but that needs to be discussed based on previous literature.

In the description of the programme I lack one piece of information: What about the offenders? They obviously did not participate at the centre, but the reader notes the high security precautions with fences etc. One gets the impression that there may be real dangers around this. And if it is dangerous during those treatment periods it is likely to be dangerous at home as well. I also realise that some of the patients might flee it there are no precautions against that. On the whole, a description of communication with responsible caregivers would help the reader.

In the inclusion/excusion criteria there is one that draws my attention:

"Be psychologically minded and able to benefit from therapy"

This seems like a soft criterion that could enfathom anything including, “I like this person”. I know it is impossible to treat people who are hostile but still, this is also something that should be discussed. In the end it would reveal what proportion of children with these problems that the treatment programme would accept. If possible, one wonders what percentage of considered patients what was accepted.

Reviewer #2: Thank you for the opportunity to review this manuscript. The authors examined the impact of a one-year multimodal treatment program for survivors of child sexual abuse (CSA), an important area of research. While the topic is meaningful and the study has the potential to contribute to the literature, several issues currently limit the clarity, coherence, and overall contribution of the manuscript.

Overall, the paper would benefit from improved organization and clearer articulation of key decisions. The introduction at times feels unfocused, with some content (e.g., prevalence estimates and Canada-specific context) appearing somewhat disconnected from the study’s aims, while other important areas—such as a more detailed overview of the treatment approaches (particularly the Four Stories framework)—require further development. Streamlining and strengthening the narrative would help situate the study more effectively.

The most significant concerns relate to the methods section. Important details are either missing or insufficiently described, including clearer justification for the inclusion of specific variables, the role of certain constructs (e.g., ACEs), and how the various therapeutic components fit together conceptually and analytically. Additional information on the measures (e.g., scoring, response options, and psychometric properties) is needed to support interpretability. Greater clarity is also required regarding the statistical analysis plan. In particular, the use of multiple t-tests raises concerns given the likely intercorrelations among outcome variables; alternative approaches (e.g., multivariate analyses) may be more appropriate. Clarification of the analytic strategy, including software used and handling of key variables, would strengthen confidence in the findings. The absence of a control or comparison group should also be more explicitly acknowledged.

In the results section, there is some redundancy between the text and tables, and opportunities to streamline presentation (e.g., summarizing rather than repeating table values). Greater clarity in figures and tables (including consistent terminology and clear labeling of acronyms) would improve readability.

The discussion would benefit from closer alignment with the study’s actual data and analyses. At times, the authors interpret findings or emphasize factors (e.g., treatment setting, caregiver involvement, and the Four Stories framework) that were not directly measured or tested, which weakens the strength of the conclusions. Tightening this section to focus on what can be directly supported by the data would enhance the manuscript’s credibility.

Finally, there are a number of minor issues related to clarity, grammar, and consistency (e.g., tense – sometimes past, sometimes present, spelling (e.g. behavior/behaviour), and typographical errors) that should be addressed throughout.

In summary, this study addresses an important topic and presents potentially valuable findings. However, revisions—particularly to the methods, analytic rationale, and alignment between aims, analyses, and interpretation—are needed to strengthen the manuscript and clarify its contribution to the field.

Reviewer #3: This study concerns a development and evaluation of a multimodal treatment program for survivors of child sexual abuse. The article is well-written and concerns an interesting and important topic.

Some remarks

Abstract

Minor: the sentence ”Participants whose PTSD scores remained above the clinical threshold at graduation were had

higher levels of family dysfunction”: contains an extra word ”were had”.

Material and methods

• This study contains a "second level independent analysis", which makes one wonder whether there is a 1st level analysis reported elsewhere?

• The screening of participants was a bit unclear, was this done by psychologists? It could be good to clarify.

• In the limitations, the authors mention that caregiver data is missing. It would have been good to also mention the risk that the accompanying parent was the abuser him/herself, was that prevented in any way?

Discussion and conclusion

* Here, it would have been good with some mentioning of how the results could perhaps be generalized to other types of less intensive treatments?

Reviewer #4: This manuscript addresses an important and difficult clinical area, and overall it reads as a solid, potentially valuable paper. The topic is relevant, the program description is detailed, and the authors appear committed to improving care for a highly vulnerable population. At the same time, in its current form the manuscript has several conceptual, methodological, and editorial issues that should be addressed before publication.

My main concern is that the paper at times overstates what can be concluded from the design. This is an uncontrolled pre-post evaluation of a residential program, apparently without preregistration. For a secondary outcome analysis of this kind, the absence of preregistration is an important limitation. More importantly, without a control group, the results cannot be interpreted as evidence of efficacy in a causal sense. Improvements may reflect treatment effects, but they may also reflect passage of time, regression to the mean, expectancy effects, environmental containment, or natural recovery. Therefore, language such as “effective” or claims implying confirmation of efficacy should be substantially softened throughout the manuscript. Relatedly, the statement that a relatively large sample and large effect sizes compensate for the lack of a control group is methodologically incorrect; effect sizes in uncontrolled designs do not establish causality.

The abstract states that “no gold standard currently exists,” yet the Introduction reportedly describes TF-CBT as a gold standard treatment. There is also an error (“were had higher levels”). Finally, the lack of a control group should already be acknowledged in the abstract.

The Introduction is generally concise and well structured. However, some sections feel overly local or insufficiently integrated for an international readership (discussion of Canadian legal terminology reform and Canadian prevalence data as the only national epidemiological context). The section on AI and CSAM is potentially interesting, but currently reads as underdeveloped and has no references.

The final hypothesis refers to “long-term results,” which is misleading if the program lasts one year and no follow-up data were collected.

The Methods section raises several concerns regarding sampling and internal validity. Inclusion criteria such as being “psychologically minded and able to benefit from therapy” are highly subjective and not operationalized; the manuscript should clarify who assessed this and according to what criteria. Similarly, IQ >80 based on “clinical assessment” is too vague. A more serious issue is that completion of the full program appears to be required for inclusion in the analysis. This creates a classic completion bias: only those who successfully completed treatment are analyzed. Since 52 participants (253 to 201) did not complete the program, the paper should report dropout analyses comparing completers vs non-completers on baseline variables. Without this, results may be substantially biased and this should be explicitly discussed in the limitations.

The intervention itself is described in commendable detail. However, I could not identify whether the program was manualized or whether treatment fidelity was monitored.

Several measurement descriptions also need correction. KIDSCREEN-10 and CRAFFT reportedly contain placeholder citations (“REFS”). CYRM-12 is described as a 17-item measure, while its name suggests 12 items. Reliability coefficients are missing of all measures.

In the Results section, a power analysis is missing. Given subgroup sizes of n=85 and n=116 and the number of tested variables, this should be addressed. More than half of participants (55.7%) received pharmacological treatment, yet no attempt appears to have been made to control for medication effects. Changes in PTSD, depression, or related outcomes may therefore be partly pharmacological rather than attributable to the program alone. Heading of Table 4 reportedly lists n=116 (Adolescent Program) although the table contains data from both programs.

The Discussion would benefit from a more cautious interpretation of findings. At present, the conclusions seem stronger than warranted by the design. This study can reasonably be framed as showing promising pre-post improvements and feasibility in a real-world residential setting, but not definitive treatment efficacy.

The limitations section is commendably transparent in acknowledging several key issues (lack of control group, self-report data, absence of caregiver data). However, additional limitations should be included: completion bias, lack of follow-up, possible confounding by medication, absence of manualization/fidelity checks, single-site design, and possible social desirability bias in self-report data collected in a residential context with staff involvement. If I understand correctly, the authors are also evaluating their own program within a single center; if so, potential conflicts of interest or allegiance effects should be transparently disclosed.

The Data Availability Statement is unclear. It is not evident why de-identified data could not be shared, at least through controlled or open access, if they have been properly anonymized. The authors should clarify the specific barriers to data sharing.

In summary, this is a worthwhile manuscript on an important topic, but the current version requires revision.

**********

6. PLOS authors have the option to publish the peer review history of their article (what does this mean?). If published, this will include your full peer review and any attached files.

If you choose “no”, your identity will remain anonymous but your review may still be made public.

**Do you want your identity to be public for this peer review?**  For information about this choice, including consent withdrawal, please see our Privacy Policy.

Reviewer #1: **Yes:** Tores Theorell Karolinska Institutet, Stockholm, Sweden

Reviewer #2: No

Reviewer #3: No

Reviewer #4: **Yes:** Monika O. Jańczak

**********

[NOTE: If reviewer comments were submitted as an attachment file, they will be attached to this email and accessible via the submission site. Please log into your account, locate the manuscript record, and check for the action link "View Attachments". If this link does not appear, there are no attachment files.]

To ensure your figures meet our technical requirements, please review our figure guidelines: https://journals.plos.org/plosone/s/figures

You may also use PLOS’s free figure tool, NAAS, to help you prepare publication quality figures: https://journals.plos.org/plosone/s/figures#loc-tools-for-figure-preparation.

NAAS will assess whether your figures meet our technical requirements by comparing each figure against our figure specifications.

# Author comment: Author response to Decision Letter 1

**DOI:** 10.1371/journal.pone.0354692.r002

21 Jun 2026

Thank you for taking the time to review this manuscript and for your insightful and helpful comments. We hope that this iteration meets the journal's requirements and has addressed all comments and concerns. Please see the attached document with responses to reviewer comments.

Attachment Submitted filename: Response to Reviewers.docx

# Peer review: Decision Letter 1

**DOI:** 10.1371/journal.pone.0354692.r003

**Authors:** Vincenzo De Luca

6 Jul 2026

PONE-D-26-10574R1

Evaluation of outcomes in 201 child and youth survivors of child sexual abuse (CSA) who participated in a one-year multimodal treatment program

PLOS One

Dear Dr. Reeson,

Thank you for submitting your manuscript to PLOS ONE. After careful consideration, we feel that it has merit but does not fully meet PLOS ONE’s publication criteria as it currently stands. Therefore, we invite you to submit a revised version of the manuscript that addresses the points raised during the review process.

Please submit your revised manuscript by Aug 20 2026 11:59PM. If you will need more time than this to complete your revisions, please reply to this message or contact the journal office at plosone@plos.org. When you're ready to submit your revision, log on to https://www.editorialmanager.com/pone/ and select the 'Submissions Needing Revision' folder to locate your manuscript file.

Please include the following items when submitting your revised manuscript:

- A letter that responds to each point raised by the academic editor and reviewer(s). You should upload this letter as a separate file labeled 'Response to Reviewers'.
- A marked-up copy of your manuscript that highlights changes made to the original version. You should upload this as a separate file labeled 'Revised Manuscript with Track Changes'.
- An unmarked version of your revised paper without tracked changes. You should upload this as a separate file labeled 'Manuscript'.

If you would like to make changes to your financial disclosure, please include your updated statement in your cover letter. Guidelines for resubmitting your figure files are available below the reviewer comments at the end of this letter.

If applicable, we recommend that you deposit your laboratory protocols in protocols.io to enhance the reproducibility of your results. Protocols.io assigns your protocol its own identifier (DOI) so that it can be cited independently in the future. For instructions see: https://journals.plos.org/plosone/s/submission-guidelines#loc-laboratory-protocols. Additionally, PLOS ONE offers an option for publishing peer-reviewed Lab Protocol articles, which describe protocols hosted on protocols.io. Read more information on sharing protocols at https://plos.org/protocols?utm_medium=editorial-email&utm_source=authorletters&utm_campaign=protocols.

As the corresponding author, your ORCID iD is verified in the submission system and will appear in the published article. PLOS supports the use of ORCID, and we encourage all coauthors to register for an ORCID iD and use it as well. Please encourage your coauthors to verify their ORCID iD within the submission system before final acceptance, as unverified ORCID iDs will not appear in the published article. *Only*  the individual author can complete the verification step; PLOS staff *cannot*  verify ORCID iDs on behalf of authors.

We look forward to receiving your revised manuscript.

Kind regards,

Vincenzo De Luca

Academic Editor

PLOS One

Journal Requirements:

1. If the reviewer comments include a recommendation to cite specific previously published works, please review and evaluate these publications to determine whether they are relevant and should be cited. There is no requirement to cite these works unless the editor has indicated otherwise.

2. Please review your reference list to ensure that it is complete and correct. If you have cited papers that have been retracted, please include the rationale for doing so in the manuscript text, or remove these references and replace them with relevant current references. Any changes to the reference list should be mentioned in the rebuttal letter that accompanies your revised manuscript. If you need to cite a retracted article, indicate the article’s retracted status in the References list and also include a citation and full reference for the retraction notice.

[Note: HTML markup is below. Please do not edit.]

Reviewers' comments:

Reviewer's Responses to Questions

**Comments to the Author**

1. If the authors have adequately addressed your comments raised in a previous round of review and you feel that this manuscript is now acceptable for publication, you may indicate that here to bypass the “Comments to the Author” section, enter your conflict of interest statement in the “Confidential to Editor” section, and submit your "Accept" recommendation.

Reviewer #1: All comments have been addressed

**********

2. Is the manuscript technically sound, and do the data support the conclusions?

The manuscript must describe a technically sound piece of scientific research with data that supports the conclusions. Experiments must have been conducted rigorously, with appropriate controls, replication, and sample sizes. The conclusions must be drawn appropriately based on the data presented.

Reviewer #1: Yes

**********

3. Has the statistical analysis been performed appropriately and rigorously?

Reviewer #1: Yes

**********

4. Have the authors made all data underlying the findings in their manuscript fully available?

The PLOS Data policy requires authors to make all data underlying the findings described in their manuscript fully available without restriction, with rare exception (please refer to the Data Availability Statement in the manuscript PDF file). The data should be provided as part of the manuscript or its supporting information, or deposited to a public repository. For example, in addition to summary statistics, the data points behind means, medians and variance measures should be available. If there are restrictions on publicly sharing data—e.g. participant privacy or use of data from a third party—those must be specified.

Reviewer #1: No

**********

5. Is the manuscript presented in an intelligible fashion and written in standard English?

PLOS ONE does not copyedit accepted manuscripts, so the language in submitted articles must be clear, correct, and unambiguous. Any typographical or grammatical errors should be corrected at revision, so please note any specific errors here.

Reviewer #1: Yes

**********

6. Review Comments to the Author

Please use the space provided to explain your answers to the questions above. You may also include additional comments for the author, including concerns about dual publication, research ethics, or publication ethics. (Please upload your review as an attachment if it exceeds 20,000 characters)

Reviewer #1: This is a useful and extensive evaluation of a treatment program. The limitations have better described in this version

I react a little to the repeated use of “reliability” for Cronbach alpha which is merely a measure of internal consistency among items included in a composite measure.

The authors should preferably say internal consistency or merely alpha. In addition, If one asks approximately the same question with small variations in formulation one can get a very high alpha which gives the reader a false impression of fantastic reliability. Reliability could be a numer of other things such as repeatability

In the very last sentence there is a spelling error… furhter…. Instead of further…

In the second paragraph of the discussion one sentence is incomplete. Something should be added

….The results of this study suggest that less intensive

treatment options that prioritize trauma-informed programming and with an emphasis on

establishing a safe environment to reattune dysregulated nervous system activation.

Apart from these small comments it is ok

**********

7. PLOS authors have the option to publish the peer review history of their article (what does this mean?). If published, this will include your full peer review and any attached files.

If you choose “no”, your identity will remain anonymous but your review may still be made public.

**Do you want your identity to be public for this peer review?**  For information about this choice, including consent withdrawal, please see our Privacy Policy.

Reviewer #1: **Yes:** Tores Theorell

**********

[NOTE: If reviewer comments were submitted as an attachment file, they will be attached to this email and accessible via the submission site. Please log into your account, locate the manuscript record, and check for the action link "View Attachments". If this link does not appear, there are no attachment files.]

To ensure your figures meet our technical requirements, please review our figure guidelines: https://journals.plos.org/plosone/s/figures

You may also use PLOS’s free figure tool, NAAS, to help you prepare publication quality figures: https://journals.plos.org/plosone/s/figures#loc-tools-for-figure-preparation.

NAAS will assess whether your figures meet our technical requirements by comparing each figure against our figure specifications.

# Author comment: Author response to Decision Letter 2

**DOI:** 10.1371/journal.pone.0354692.r004

8 Jul 2026

Please see attached document "Response to Reviewers (2)"

Figure 3 has been linked in the text

Figure 3 caption added to the text

Attachment Submitted filename: Response_to_Reviewers_auresp_2.docx

# Peer review: Decision Letter 2

**DOI:** 10.1371/journal.pone.0354692.r005

**Authors:** Vincenzo De Luca

13 Jul 2026

Evaluation of outcomes in 201 child and youth survivors of child sexual abuse (CSA) who participated in a one-year multimodal treatment program

PONE-D-26-10574R2

Dear Dr. Reeson,

We’re pleased to inform you that your manuscript has been judged scientifically suitable for publication and will be formally accepted for publication once it meets all outstanding technical requirements.

Within one week, you’ll receive an e-mail detailing the required amendments. When these have been addressed, you’ll receive a formal acceptance letter and your manuscript will be scheduled for publication.

An invoice will be generated when your article is formally accepted. Please note, if your institution has a publishing partnership with PLOS and your article meets the relevant criteria, all or part of your publication costs will be covered. Please make sure your user information is up-to-date by logging into Editorial Manager at Editorial Manager® and clicking the ‘Update My Information' link at the top of the page. For questions related to billing, please contact billing support.

If your institution or institutions have a press office, please notify them about your upcoming paper to help maximize its impact. If they’ll be preparing press materials, please inform our press team as soon as possible -- no later than 48 hours after receiving the formal acceptance. Your manuscript will remain under strict press embargo until 2 pm Eastern Time on the date of publication. For more information, please contact onepress@plos.org.

Kind regards,

Vincenzo De Luca

Academic Editor

PLOS One

Additional Editor Comments (optional):

Reviewers' comments:

Reviewer's Responses to Questions

**Comments to the Author**

1. If the authors have adequately addressed your comments raised in a previous round of review and you feel that this manuscript is now acceptable for publication, you may indicate that here to bypass the “Comments to the Author” section, enter your conflict of interest statement in the “Confidential to Editor” section, and submit your "Accept" recommendation.

Reviewer #1: All comments have been addressed

**********

2. Is the manuscript technically sound, and do the data support the conclusions?

The manuscript must describe a technically sound piece of scientific research with data that supports the conclusions. Experiments must have been conducted rigorously, with appropriate controls, replication, and sample sizes. The conclusions must be drawn appropriately based on the data presented.

Reviewer #1: Yes

**********

3. Has the statistical analysis been performed appropriately and rigorously?

Reviewer #1: Yes

**********

4. Have the authors made all data underlying the findings in their manuscript fully available?

The PLOS Data policy requires authors to make all data underlying the findings described in their manuscript fully available without restriction, with rare exception (please refer to the Data Availability Statement in the manuscript PDF file). The data should be provided as part of the manuscript or its supporting information, or deposited to a public repository. For example, in addition to summary statistics, the data points behind means, medians and variance measures should be available. If there are restrictions on publicly sharing data—e.g. participant privacy or use of data from a third party—those must be specified.

Reviewer #1: No

**********

5. Is the manuscript presented in an intelligible fashion and written in standard English?

PLOS ONE does not copyedit accepted manuscripts, so the language in submitted articles must be clear, correct, and unambiguous. Any typographical or grammatical errors should be corrected at revision, so please note any specific errors here.

Reviewer #1: Yes

**********

6. Review Comments to the Author

Please use the space provided to explain your answers to the questions above. You may also include additional comments for the author, including concerns about dual publication, research ethics, or publication ethics. (Please upload your review as an attachment if it exceeds 20,000 characters)

Reviewer #1: You have taken into account the reviewer comments. I therefore recommend publication.

In particular I noticed your expansion of the discussion regarding lack of control group

**********

7. PLOS authors have the option to publish the peer review history of their article (what does this mean?). If published, this will include your full peer review and any attached files.

If you choose “no”, your identity will remain anonymous but your review may still be made public.

**Do you want your identity to be public for this peer review?**  For information about this choice, including consent withdrawal, please see our Privacy Policy.

Reviewer #1: **Yes:** Tores Theorell

**********

# Editor report: Acceptance letter

**DOI:** 10.1371/journal.pone.0354692.r006

**Authors:** Vincenzo De Luca

PONE-D-26-10574R2

PLOS One

Dear Dr. Reeson,

I'm pleased to inform you that your manuscript has been deemed suitable for publication in PLOS One. Congratulations! Your manuscript is now being handed over to our production team.

At this stage, our production department will prepare your paper for publication. This includes ensuring the following:

* All references, tables, and figures are properly cited

* All relevant supporting information is included in the manuscript submission,

* There are no issues that prevent the paper from being properly typeset

You will receive further instructions from the production team, including instructions on how to review your proof when it is ready. Please keep in mind that we are working through a large volume of accepted articles, so please give us a few days to review your paper and let you know the next and final steps.

Lastly, if your institution or institutions have a press office, please let them know about your upcoming paper now to help maximize its impact. If they'll be preparing press materials, please inform our press team within the next 48 hours. Your manuscript will remain under strict press embargo until 2 pm Eastern Time on the date of publication. For more information, please contact onepress@plos.org.

You will receive an invoice from PLOS for your publication fee after your manuscript has reached the completed accept phase. If you receive an email requesting payment before acceptance or for any other service, this may be a phishing scheme. Learn how to identify phishing emails and protect your accounts at https://explore.plos.org/phishing.

If we can help with anything else, please email us at customercare@plos.org.

Thank you for submitting your work to PLOS ONE and supporting open access.

Kind regards,

PLOS ONE Editorial Office Staff

on behalf of

Dr. Vincenzo De Luca

Academic Editor

PLOS One
